# The efficacy and medium-term outcomes of ligament advanced reinforcement system compared with auto-grafts in anterior cruciate ligament reconstruction: At least 2 years follow-up

**DOI:** 10.3389/fbioe.2022.960075

**Published:** 2022-09-02

**Authors:** Bingxian Ma, Yongxiang Wang, Yongsheng Xu

**Affiliations:** Department of Orthopedics, Inner Mongolia People’s Hospital, Hohhot, China

**Keywords:** anterior cruciate ligament reconstruction (ACLR), ligament advanced reinforcement system (LARS), artificial ligament, biomaterials, biomechanical property, knee stability

## Abstract

**Background:** Graft choice is an important step in the pre-operative plan of anterior cruciate ligament reconstruction (ACLR). The four-strand hamstring tendon (4SHT) is the most widely used auto-graft, while the Ligament Advanced Reinforcement System (LARS) is the newest typical biomaterial for ACLR. The physical activity level (PAL) before injury can affect the efficacy and outcomes of ACLR. This study aims to compare the efficacy and functional outcomes between ACLR using LARS and 4SHT in patients different PALs.

**Methods:** This was a prospective paired case-control study. ACL rupture patients included from 1 January 2017 to 31 December 2019 were subsequently divided into the high and plain PAL groups, according to their baseline PAL before injury. Clinical assessments included: Lachman test, pivot shift test, ligament laxity, Lysholm and International Knee Documentation Committee (IKDC) scores, and rate of returning to sports. The minimum follow-up was 2 years (y).

**Results:** A total of 58 patients had accomplished the 2 y follow-up (missing rate: 6.5%). In the high PAL group (n = 22), the positive rate of A–P laxity of the LARS subgroup was lower than the 4SHG subgroup (*p* = 0.138), while the Lysholm score (*p* = 0.002), IKDC score (*p* = 0.043), and rate of returning to sports (*p* = 0.010) of the LARS were higher than the 4SHG at 1 year follow-up; the positive rates of A–P laxity (*p* = 0.009) and pivot test (*p* = 0.027) were lower in the LARS than the 4SHG at 2 y follow-up. In the plain PAL group (n = 36), the positive rate of A–P laxity in the LARS subgroup was lower than the 4SHG at 1 year follow-up (*p* = 0.017); the positive rates of A–P laxity (*p* = 0.001), Lachman (*p* = 0.034), and pivot tests (*p* = 0.034) in the LARS were also lower than the 4SHG at 2 y follow-up, but the IKDC score (*p* = 0.038) and rate of returning to sports (*p* = 0.019) in the 4SHG were higher than the LARS.

**Conclusion:** In patients with high PAL, LARS can acquire better knee stability, sooner functional recovery, and returning to sports than 4SHG, while in patients without high PAL, 4SHG acquires better functional outcomes and a higher rate of returning to sports.

## Introduction

Anterior cruciate ligament (ACL) injury is a very common disease in sports medicine, and it occurs both in athletes and common people. In order to restore knee function and sports ability, arthroscopic ACL reconstruction (ACLR) is performed in ACL rupture patients in most cases. ACLR always needs careful pre-operative planning, according to the differences in patients’ background, including the career, baseline physical activity level (PAL), age, and returning to sports. The graft choice is a very important step of the operation strategy because the graft quality and characteristics are directly associated with the clinical efficacy and outcome of ACLR (Moghamis et al., 2019).

The present clinically used grafts for ACLR include auto-grafts, artificial grafts, and allogeneic tendon grafts, each one has its unique characteristics and advantages (Yang et al., 2020). Double-bundle four-strand hamstring tendon (4SHT) is the most commonly used auto-grafts for ACLR (Gifstad et al., 2013), and evidence have suggested that it can provide better anterior–posterior (A–P) stability and functional outcomes (Yang et al., 2020). Ligament Advanced Reinforcement System (LARS) is a typical scaffold-type biomaterial for ligament reconstruction, produced by the newest synthetic technique (Jia et al., 2017). The LARS ligament is manufactured by polyethylene terephthalate and consists of two different parts (Parchi et al., 2013): ① the intra-osseous part is made of longitudinal fibers, bound together by a transverse knitted structure; ② the intra-articular part comprises only longitudinal parallel fibers, which are pre-twisted at 90°. The stretching resistance force of LARS is much stronger than an intact ACL. It has been reported that LARS can accelerate the rehabilitation and fasten the early return to sports, attributed by the refined biomaterial quality (Krupa et al., 2016; Bugell et al., 2017).

Until now, there is still controversy about which kind of graft is the best choice for ACLR (Su et al., 2020). We consider that the patient’s baseline physical activity may have impacts on the efficacy and outcomes of ACLR, consequently affecting the result of a graft choice. Although several clinical studies have compared the efficacy of ACLR using 4SHT with using LARS, few has focused on the differences in the patients’ physical exercise background or career, lacking of systematic comparisons performed in the subgroups of different activity levels. We hypothesized that ACLR using 4SHT or LARS may have the different results of efficacy and functional outcomes in patients with different PALs.

## Materials and methods

### Patient involvement

Patients diagnosed with ACL rupture were included between 1 January 2017 and 31 December 2019, in our department. The inclusion criteria were: ① age: 20–50 years old, BMI≤31; ② acute ACL rupture less than 3 months; ③ single knee; ④ agree to participate in this study after signing the informed consent; ⑤ agree to the arrangement of the subgrouping (graft choice). The exclusion criteria were: ① multi-ligament injury; ② ACL re-rupture; ③ history of lower extremity fracture, ligament rupture, and operations; ④ knee osteoarthritis with the Kellgren–Lawrence grade>2, rheumatoid arthritis, and gouty arthritis; ⑤ nerve system diseases such as Parkinson’s disease.

The protocols and procedures for the protection of human subjects reviewed and approved by the Ethics Committee of Inner Mongolia People’s Hospital.

### PAL levels and grouping

In order to evaluate patients’ PAL before the injury (in the past 6 months), they were asked for their weekly physical exercise intensity and duration, and the International Physical Activity Questionnaire (IPAQ) was used to assess the PAL (Ethnic et al., 2009). IPAQ has been used for assessing the pre-operative PAL in the field of surgery (Angenete et al., 2016), it was developed as a common questionnaire to measure multiple domains of physical activity in all countries, including the leisure time, work, transportation, and house-hold tasks (Ethnic et al., 2009), which is particularly important in the developing and transitional countries because where assessment confined to leisure time activity may miss substantial daily physical activity undertaken for the purpose of work or travel (Bull et al., 2004). High PAL includes professional athletes, sports enthusiasts, and common people who meet either of the two criteria of high level from the International Physical Activity Questionnaire (IPAQ) (Ethnic et al., 2009): ① 40 min of vigorous-intensity activity such as jogging, lap swimming, and playing tennis >3 days/week; ② 60 min of moderate-intensity activity such as brisk walking, casual bicycling, and gardening >5 days a week.

This was a prospective paired cohort study. Patients with high PAL were equally divided into the 4HSG and LARS subgroups after matching their sex and age, and patients with plain PAL were also divided into the two subgroups after matching.

### Arthroscopic ACLR with different grafts

Routine arthroscopy and anatomic ACLR were performed on all patients by the same senior surgeon, and any meniscal and/or cartilaginous injuries were treated before ACLR.

In the 4SHG subgroup, both the semitendinosus tendon and gracilis tendon were harvested and disconnected, folded into four strands, and sutured to each other. The tibial drill guide was adjusted to a sagittal angle of 55°, and the intra-articular guide tip was placed in the center of the ACL stump, which was preserved as much as possible. The femoral tunnel guide tip was located at the ACL femoral insertion site. The femoral tunnel diameter ranged from 7.5 to 8.0 mm, according to the diameter of the auto-graft. The femoral side was fixed with an Endobutton (Smith & Nephew, Memphis, TN, United States), and the tibial side was fixed with a bio-absorbable (poly-l-lactic acid) interference screw (Biosure HA; Smith & Nephew).

In the LARS (LARS; Surgical Implants and Devices, Arc-sur-Tille, France) subgroup, the intra-articular guiding of ACL tibial and femoral insertion site was the same, and the ACL stump was also preserved as much as possible. Diameters of the tibial and femoral bone tunnel were set at 7.5 or 8.0 mm, which matched with the chosen graft, respectively. If the patients’ weight was over 80 kg, we chose the AC50DB (8.0 mm, 100- gauge fiber), otherwise the AC40DB (7.5 mm, 80-gauge fiber). LARS ligament was introduced into the joint from the tibial tunnel by a guidewire. The tension was adjusted by a full range of motion (ROM) of the knee, and no impingement was found. Both the tibial and femoral fixations were performed with the titanium interference-fit screws (Surgical Implants and Devices; LARS).

### Rehabilitation

Both groups underwent the same rehabilitation scheme. The isometric contraction of the quadriceps was started as soon as possible after surgery. A lockable functional brace was used for a whole day in the first 2 weeks, and it was only used during walking for the next 2 weeks. A ROM of 90° could be achieved in the first week, and 120° in 8 weeks. For muscle strength, patients were requested to do the leg-raising exercises with the brace at least 200 times/day, during the first month after surgery. Full weight-bearing (walking without crutches) was allowed at least 1 month after surgery.

### Follow-ups

The follow-up was started when ACLR was completed. The end was re-rupture/death/missing, whichever occurred first. The minimum follow-up was 1 year (y), and the maximum was 2 y. General clinical parameters included: the injury time before operation, meniscus tear (yes/no), diameter of the graft, follow-up time, and complications. The clinical assessments of the efficacy and functional outcomes of ACLR included: the physical examination, ligament laxity (KT-2000), and knee ROM, as well as the subjective scoring systems of knee function. Knee X-ray photographs were also performed at 1 and 2 y follow-ups to evaluate the internal fixation location of the reconstructed ACL.

All of the follow-up data were checked and entered into a database by two researchers, and a double-entry is carried out for the quality control.

### Clinical assessments

Lachman test and pivot shift test are two physical examinations that can determine ACL rupture, which can be used to determine the stability recovery after ACLR (Yang et al., 2020). Lachman test was used to assess the A–P joint stability, and pivot shift test was used to assess the rotational stability (Cui et al., 2022). Lachman test was classified as: hard end-point (-), doubtable laxity (±), and soft end-point (+). Pivot shift was classified as (Bull et al., 2004): normal (-), glide (±), and clunk or gross (+).

Ligament laxity was measured by: the forward shift is tested when the knee is flexed at 30°. A–P laxity is evaluated by comparing to the healthy side, and it is classified as (Ranger et al., 2011): normal, grade 1 (difference between 1 and 5 mm), grade 2 (between 5 and 10 mm), and grade 3 (>10 mm).

The knee flexion contracture (KFC) angle was assessed by passive physical examination of ROM. KFC is defined as the gap value of extension loss compared to the normal side, and more than 5° was considered as KFC, according to the Knee Society Score (KSS) system (Insall et al., 1989; Yi et al., 2020). KFC degree is classified as normal (<5°), grade 1 (mild, KFC between 5° and 15°), grade 2 (moderate, between 15° and 30°), and grade 3 (severe, KFC >30°) (Insall et al., 1989; Yi et al., 2020).

### Subjective assessments of knee function

To evaluate the functional outcomes of living quality and motor function, the Lysholm knee scoring scale (Wang et al., 2016) and International Knee Documentation Committee (IKDC) score (Fu and Chan, 2011) were accessed by self-questionnaires at follow-ups. The rate of returning to sports was used to evaluate the outcome of physical activity.

### Clinical failure

At 2 y follow-up, cases who met any of the following results were regarded as clinical failure (Su et al., 2020): ① an overall IKDC score of C (60–70 score) or D (<60–70 score); ② Lachman test (+); ③ pivot shift test (+); ④ A–P laxity of grade 2 or 3; ⑤ KFC; and ⑥ re-rupture. The clinical failure rates were calculated.

### Statistical analysis

Continuous data were expressed as mean ± SD, and comparisons of the continuous data were processed by the independent samples t-tests and Levene variance homogeneity tests between subgroups. Count data were expressed as number (n) and rate (/), and comparisons of the count data were processed by the Chi-square test or Fisher’s exact test. The level of significance was set at 0.05. All of the statistical analyses were performed using SPSS 20.0 (SPSS Inc, 2009; Chicago, IL, United States).

## Results

### Basic characteristics

Finally, 58 patients had accomplished the 2 y follow-up. The high PAL group (n = 22) consisted of 11 professional athletes, six college students, two military soldiers, one manual worker, and two sports teachers/coaches. The plain PAL group (n = 36) consisted of 23 office workers, seven college students, three high school students, and three freelancers/housewives. The patients of the two groups were divided equally into the 4SHG and LARS subgroups after matching their sex and age. Most of the patients were injured by sports (n = 52): 35 subjects suffered a sprain of the knee when doing competitive sports, 17 sprained the knee by themselves when skiing or skating; four slipped and sprained the knee by themselves during farming, and two were caused by motor vehicle accident (n = 6). The basic characteristics, as well as baseline medical characteristics of the 4SHG and LARS subgroups, did not have significance in the two groups (Table 1).

**TABLE 1:** Basic characteristics of the 4SHG and LARS subgroups in patients with different PALs.

Characteristics	High PAL (n = 22)			Plain PAL (n = 36)		
	4SHG	LARS	*p*-value	4SHG	LARS	*p*-value
Enrolled subjects (n)	11	11	*--*	18	18	--
Sex (male/female)	7/4	8/3	*χ* ^2^ = 0.210	11/7	9/9	*χ* ^2^ = 0.450
			*p* = 0.647			*p* = 0.502
Age (year)	25.4 ± 7.2	25.8 ± 6.5	*t* = -0.125	33.4 ± 8.9	33.5 ± 7.8	*t* = -0.040
			*p* = 0.902			*p* = 0.968
BMI	22.05 ± 1.16	22.83 ± 1.54	*t* = -1.345	24.21 ± 1.86	24.00 ± 1.63	*t* = 0.361
			*p* = 0.194			*p* = 0.720
Time before ACLR (week)	3.7 ± 4.4	2.4 ± 3.4	*t* = 0.812	3.6 ± 4.1	4.0 ± 4.7	*t* = -0.282
			*p* = 0.426			*p* = 0.780
Meniscus injury (with/without)	3/8	2/10	*χ* ^2^ = 0.259	5/13	5/13	*--*
			*p* = 0.611			
Graft diameter (mm)	7.86 ± 0.23	7.82 ± 0.25	*t* = 0.439	7.86 ± 0.23	7.86 ± 0.23	*--*
			*p* = 0.666			

PAL (physical activity level), BMI (body mass index), ACLR (anterior cruciate ligament reconstruction).

### Follow-ups

At the beginning of this study, there were 62 patients included in this study, and the initial sample size of the high PAL group and plain PAL group were 22 and 40, respectively. The patients of the two groups were divided equally into the 4SHG and LARS subgroups after matching their sex and age. One patient of the plain PAL group (4SHG subgroup) was missing at the 1 year follow-up, and another three patients of the plain PAL group (1 in 4SHG subgroup, two in LARS subgroup) were missing at the 2 y follow-up. The four missing patients cannot be contacted through phone call/e-mail or declaimed that he/she cannot participate anymore. The total missing rate was 6.5% (4/62). Those missing subjects were excluded from the database, in order to control the bias.

### Comparisons of 4SHG and LARS in patients with high PAL

In the high PAL group, the positive rates of Lachman and pivot shift test, as well as KFC did not have significance between the 4SHG subgroup and LARS subgroup at 1 year follow-up. However, the positive rate of A–P shift in the 4SHG subgroup was higher than in the LARS subgroup, and the Lysholm score, IKDC score, as well as the rate of returning to sports in the 4SHG subgroup was lower than the LARS subgroup at 1 year follow-up (Table 2). At 2 y follow-up, the positive rates of Lachman and KFC still did not have significance between the subgroups, but the positive rates of pivot shift and A–P shift in the 4SHG subgroup were higher than those in the LARS subgroup; however, the Lysholm score, IKDC score, and rate of returning to sports did not show a significant difference between the two subgroups (Table 2). At 2 y follow-up, only three clinical failure cases with the A–P laxity of grade 2 were found in the 4SHG subgroup; however, the failure rate did not show a significant difference between subgroups (Table 2).

**TABLE 2:** Comparisons of efficacy, safety, and functional outcomes between the 4SHG and LARS subgroups in high PAL patients.

Parameter		1 y follow-up (n = 22)			2 y follow-up (n = 22)		
		4SHG	LARS	*p*-value	4SHG	LARS	*p*-value
Lachman	-	11	11	*--*	8	11	*χ* ^2^ = 3.474
	±	0	0		3	0	*p* = 0.062
	+	0	0		0	0	
Pivot shift	-	9	11	*χ* ^2^ = 2.200	7	11	*χ* ^2^ = 4.899
	±	2	0	*p* = 0.138	4	0	*p* = 0.027^∗^
	+	0	0		0	0	
A–P laxity	Normal	4	10	*χ* ^2^ = 7.071	2	9	*χ* ^2^ = 9.455
	Grade 1	7	1	*p* = 0.008^∗∗^	6	2	*p* = 0.009^∗^
	Grade 2	0	0		3	0	
	Grade 3	0	0		0	0	
KFC	Normal	11	11	--	11	11	*--*
	Grade 1	0	0		0	0	
	Grade 2	0	0		0	0	
	Grade 3	0	0		0	0	
Lysholm		76.00 ± 4.70	82.64 ± 4.12	*t* = -3.513	87.27 ± 5.18	89.27 ± 4.52	*t* = -0.965
				*p* = 0.002^∗∗^			*p* = 0.346
IKDC		74.18 ± 5.31	79.09 ± 5.36	*t* = -2.159	81.91 ± 5.11	83.45 ± 3.14	*t* = -0.855
				*p* = 0.043^∗^			*p* = 0.403
Failure rate	Yes	--	--	--	3	0	*χ* ^2^ = 3.474
	No				8	11	*p* = 0.062
Returning to sports	Yes	3	9	*χ* ^2^ = 6.600	11	11	*--*
	No	8	2	*p* = 0.010^∗^	0	0	

PAL (physical activity level), A–P (anterior–posterior), KFC (knee flexion contracture); Lachman test was classified as: hard end-point (-), doubtable laxity (±), soft end-point (+); pivot shift was classified as: normal (-), glide (±), clunk or gross (+); A–P laxity was performed by KT-2000, at 30° flexion and classified as: normal, grade 1 (1–5 mm), grade 2 (5–10 mm), and grade 3 (>10 mm); KFC is classified as normal (<5°), grade 1 (5°–15°), grade 2 (15°–30°), and grade 3 (KFC >30°).

∗, *p* < 0.05.

∗∗, *p* < 0.01.

### Comparisons of 4SHG and LARS in patients with plain PAL

At 1 year follow-up, the positive rates of Lachman test, pivot shift and KFC, and the Lysholm and IKDC scores, as well as the rate of returning to sports did not have significance between the 4SHG subgroup and LARS subgroup in patients without high PAL (Table 3). The positive rate of A–P shift in the 4SHG subgroup was lower than that in the LARS subgroup at 1 year follow-up (Table 3). At 2 y follow-up, the positive rates of Lachman, pivot shift, and A–P shift in the 4SHG subgroup were higher than those in the LARS subgroup; however, the rate of returning to sports and IKDC score in the LARS subgroup were lower than that in the 4SHG subgroup (Table 3). The KFC rate and Lysholm score did not have a significant difference between the two subgroups at 2 y follow-up (Table 3). At 2 y follow-up, there were four clinical failure cases with the A–P laxity of grade 2 in the 4SHG subgroup, and one clinical failure case with KFC in the LARS subgroup, and the failure rate did not show a significant difference between subgroups (Table 3).

**TABLE 3:** Comparisons of efficacy, safety, and functional outcomes between the 4SHG and LARS subgroups in plain PAL patients.

Parameter		1 y follow-up (n = 36)			2 y follow-up (n = 36)		
		4SHG	LARS	*p*-value	4SHG	LARS	*p*-value
Lachman	-	17	18	*χ* ^2^ = 1.029	14	18	*χ* ^2^ = 4.500
	±	1	0	*p* = 0.310	4	0	*p* = 0.034^∗^
	+	0	0		0	0	
Pivot shift	-	16	18	*χ* ^2^ = 2.118	14	18	*χ* ^2^ = 4.500
	±	2	0	*p* = 0.146	4	0	*p* = 0.034^∗^
	+	0	0		0	0	
A–P shift	Normal	8	16	*χ* ^2^ = 8.121	4	15	*χ* ^2^ = 14.138
	Grade 1	9	2	*p* = 0.017^∗^	10	3	*p* = 0.001^∗^
	Grade 2	1	0		4	0	
	Grade 3	0	0		0	0	
KFC	Normal	18	16	*χ* ^2^ = 2.118	18	17	*χ* ^2^ = 1.029
	Grade 1	0	2	*p* = 0.146	0	1	*p* = 0.310
	Grade 2	0	0		0	0	
	Grade 3	0	0		0	0	
Lysholm		76.33 ± 4.58	77.39 ± 4.79	*t* = -0.676	85.56 ± 4.15	82.72 ± 4.44	*t* = 1.978
				*p* = 0.504			*p* = 0.056
IKDC		75.67 ± 3.65	78.22 ± 3.62	*t* = -1.842	86.56 ± 3.94	84.00 ± 3.09	*t* = 2.165
				*p* = 0.074			*p* = 0.038^∗^
Failure rate	Yes	--	--	*--*	4	1	*χ* ^2^ = 2.090
	No				14	17	*p* = 0.148
Returning to sport	Yes	2	4	*χ* ^2^ = 0.800	12	5	*χ* ^2^ = 5.461
	No	16	14	*p* = 0.371	6	13	*p* = 0.019^∗^

PAL (physical activity level), A–P (anterior–posterior), KFC (knee flexion contracture); Lachman test was classified as: hard end-point (-), doubtable laxity (±), soft end-point (+); pivot shift was classified as: normal (-), glide (±), clunk or gross (+); A–P laxity was performed by KT-2000, at 30° flexion and classified as: normal, grade 1 (1–5 mm), grade 2 (5–10 mm), and grade 3 (>10 mm); KFC is classified as normal (<5°), grade 1 (5°–15°), grade 2 (15°–30°), and grade 3 (KFC >30°).

∗, *p* < 0.05.

## Discussion

Recently, 4SHT and LARS have become the most commonly used auto-graft and biomaterial artificial graft for ACLR (Gifstad et al., 2013; Yang et al., 2020) (Liu et al., 2010; Su et al., 2020), respectively. Because of their different origins, 4SHT and LARS have unique characteristics and advantages (Yang et al., 2020), which was why we considered that 4SHT and LARS may have different application populations. ACL often occurs in athletes, or in common people who are doing improper exercise or fitness. The patient’s baseline PAL in pre-injury is one of the most powerful factors affecting the efficacy and outcomes of ACLR. It has been well known that athletes/sports enthusiasts who had high PAL can obtain distinct results of efficacy and outcomes, compared to the common people without high PAL. In this article, we systematically compared the ACLR using 4SHT with using LARS in two different patients with or without high PAL. The results showed that the efficacy and functional outcomes of 4SHT and LARS were not consistent in patients with different PALs, which verified the previous hypothesis.

### LARS resulted in better efficacy and outcomes in high PAL patients

In high PAL patients, ACLR with LARS can obtain better knee stability, sooner functional recovery and returning to sports than the 4SHG subgroup. The results found that the A–P stability of LARS was higher than that of 4SHG at 1 year follow-up, and the A–P stability and rotation stability were found at 2 y follow-up. LARS is a newly developed artificial ligament, unlike the earlier synthetic ligaments, it consists of two distinct segments (Parchi et al., 2013): ① the intra-osseous part, which is made of longitudinal fibers, bound together by a transverse knitted structure; ② the intra-articular part, which comprises only longitudinal parallel fibers (free fibers), pre-twisted at 90°. The architecture of LARS allows it to mimic the natural ACL and produce sufficient joint stability in short-term. Three reasons may contribute to the superior joint stability of LARS, which was found at the short-term follow-up (1 year). First, LARS is much stronger than the natural ACL or 4SHG auto-graft due to its biomechanical properties, the ultimate tensile strength of 7.5 and 8.0 mm LARS is 3,600 and 4,600 N, respectively (Bourlos et al., 2016). Second, the orientation of intra-articular free fibers helps to reduce the shearing forces. Literally, LARS can produce sufficient joint stability immediately after ACLR, correcting the dislocation as soon as possible (Ye et al., 2013). Third, LARS also works as a scaffold, which induced the fibroblastic ingrowth between the fibers (Yuanliang et al., 2020). Hence, the ACL stumps were preserved as much as we can to encourage the fibro-vascular ingrowth. *In vivo* study found that the fibroblastic ingrowth begins immediately after ACLR, which can form a sheath wrapping the intra-articular segment at 1 month post-operatively, afterward the re-growth of LARS was found at 6 months post-operatively, which presented as a completely covered ligament with regenerated collagen, synovial tissue, and vascular network, similar to the normal ACL (Yu et al., 2014). Hence, the re-growth time of LARS is shorter than that of the 4SHG auto-graft, and the latter usually costs 1 year for the vascular formation. Moreover, the ingrowth of soft tissue between the LARS fibers acts as a viscoelastic element, protecting the reconstructed ligament against the friction in bone tunnel (Vaquette et al., 2013). Those aforementioned mechanisms can explain the phenomenon of why a better joint stability can be found in the LARS subgroup at 1 year post-operatively.

The results also showed that the Lysholm score, IKDC score, and the rate of returning to sports in the LARS subgroup were higher than the 4SHG at 1 year follow-up; however, those parameters were comparable between two subgroups at 2 y follow-up. It indicated that although LARS and 4SHG resulted in similar functional outcomes eventually, ACLR using LARS can obtain a sooner recovery of knee function and returning to sports than 4SHG. It has been reported that LARS may acquire earlier symptom relief and function restoration than hamstring auto-grafts (Chen et al., 2017), and Newman et al. (2013) pointed out that the medium-term outcomes of LARS and auto-graft were comparable. The sooner recovery of LARS can be attributed by its superior efficacy of knee stability in the short-term post-operatively. As ACL is the first stabilizer of knee, ACLR using LARS results in better functional recovery in short-term. It has been reported that LARS can accelerate the rehabilitation and fasten the early return to sports, which is attributed by the refined biomaterial quality (Krupa et al., 2016; Bugell et al., 2017). Hence, LARS is very suitable for patients with high PAL, such as athletes and sports enthusiasts, who required a sooner recovery and returning to sports. LARS ligaments have been approved by CFDA since 2004 and been used for ACL reconstruction over 30,000 cases in China (Chen et al., 2019). Due to the early return to sports and impressive clinical effects, LARS has gradually become the most widely accepted biomaterial for athlete patients in China (Krupa et al., 2016; Bugell et al., 2017).

### 4SHG resulted in better functional outcomes in common patients

The results in the plain PAL patients were not consistent with the high PAL patients. The present results found that the 4SHG subgroup resulted in a higher IKDC score, as well as a higher rate of returning to sports at 2 y follow-up, comparing to the LARS. It indicated that ACLR using 4SHG could be more suitable than the LARS for common people. 4SHG consists of the auto semitendinosus tendon and gracilis tendon, and this double-bundle four-strand auto-graft can reconstruct the anteromedial and posterolateral bundle of ACL, respectively (Eck et al., 2010; Jon, 2011). Thus, 4SHG can restore the anatomical structure of natural ACL as much as possible, with fewer complications (Xie et al., 2015). LARS may not be suitable for common people. A long-term follow-up study has reported that the unsatisfied patients were over 40% after ACLR with LARS, and 27.8% of patients sustained a re-rupture, with an averaged IKDC of 76.60 ± 18.18 (Tiefenböck et al., 2015). A meta-analysis of long-term (>2 years) follow-ups also showed that the rank of Lysholm score and IKDC score in 4SHG were higher than artificial grafts including LARS, and the authors concluded that 4SHG may be a better choice for the patients who underwent ACLR (Yang et al., 2020); however, this meta-analysis did not perform the comparisons in different populations, many included study did not present the baseline PAL data, only several studies declaimed that they had included the athletes/recreational players.

In the present study, the only complication was KFC, and other complications such as infection, fever, re-rupture, or hemarthrosis were not found during follow-ups. In the LARS subgroup of plain PAL patients, two KFC cases were found at 1 y follow-up, and one case’s KFC symptom still existed at 2 y follow-up. We considered the reversible KFC case may be caused by synovitis, and the other case may be caused by a foreign body reaction. It has been reported that ACL can cause a rare foreign body reaction with granuloma (Henry et al., 2018), which blocks knee flexion by passive physical examination, causing the KFC.

Our results found that although the LARS subgroup had a higher joint stability than the 4SHG, the former resulted in a lower IKDC score and a lower rate of returning to sports at the medium-term follow-up, eventually. However, this inconsistency between knee stability and function did not happen in the high PAL patients. The differences in the patients’ baseline PAL and proprioceptive sensation may be one of the main reasons. It has been known that the proprioceptive sensation and static postural control are impaired after the injury and operation (Relph and Herrington, 2016; Kim et al., 2017) because the loss of mechanical receptors can decrease the neuromuscular control of knee stability. Patients with high PAL, such as athletes/sports enthusiasts, are prone to have a highly developed neuromuscular system and proprioceptive sensation system, which contributes to the sooner functional recovery and returning to sports after ACLR with LARS. On the contrary, in patients without high PAL, the neuromuscular system and proprioceptive sensation are very weak to adapt to the artificial ligament (LARS) during the post-operative recovery period, which leads to worse functional outcomes. It has been concluded that the non-suitable patients’ selection was one of the main failure reasons of LARS in ACLR (Chen et al., 2019). The present results suggest that the pre-injury PAL may be an indicator for the patients’ selection for ACLR using LARS.

### Limitations

Our study still has several limitations. First, the sample size of each subgroup was small. Literally, more potential affecting factors like lifestyle, job, and family commitments also needed to be matched. However, the sample size of this prospective study was no sufficient to do those matching, and we can only choose to match the main factors (sex and age) in order to control the bias. Second, the follow-up has lasted 2 y, which only presents the medium-term outcomes. Third, the present study cannot avoid the recall bias generated from self-reported IPAQ of pre-injury. Furthermore, multi-center longitudinal studies with more samples and long-term follow-ups are required to compare and determine the long-term functional outcomes of LARS and auto-grafts in patients with different PALs.

## Conclusion

The efficacy and functional outcomes of ACLR using LARS and 4SHG were not consistent in patients with different pre-injury PALs. In patients with high PAL, LARS can obtain better knee stability, sooner functional recovery and returning to sports than 4SHG, while in patients without high PAL, 4SHG acquires better functional outcomes and higher rate of returning to sports.

## Data Availability

The original contributions presented in the study are included in the article/Supplementary Material; further inquiries can be directed to the corresponding author.
